# Identification of CDH23 mutations in Korean families with hearing loss by whole-exome sequencing

**DOI:** 10.1186/1471-2350-15-46

**Published:** 2014-04-28

**Authors:** Hae-Mi Woo, Hong-Joon Park, Mi-Hyun Park, Bo-Young Kim, Joong-Wook Shin, Won Gi Yoo, Soo Kyung Koo

**Affiliations:** 1 Division of Intractable Diseases, Center for Biomedical Sciences, National Institute of Health, Chungcheongbuk-do 363-951, South Korea; 2 Soree Ear Clinic, Seoul, South Korea; 3 Division of Malaria and Parasitic Diseases, Center for Immunology and Pathology, National Institute of Health, Chungcheongbuk-do South Korea

**Keywords:** Hearing loss, *CDH23*, Mutation, Whole-exome sequencing

## Abstract

**Background:**

Patient genetic heterogeneity renders it difficult to discover disease-cause genes. Whole-exome sequencing is a powerful new strategy that can be used to this end. The purpose of the present study was to identify a hitherto unknown mutation causing autosomal recessive nonsyndromic hearing loss (ARNSHL) in Korean families.

**Methods:**

We performed whole-exome sequencing in 16 individuals from 13 unrelated small families with ARNSHL. After filtering out population-specific polymorphisms, we focused on known deafness genes. Pathogenic effects of the detected mutations on protein structure or function were predicted via *in silico* analysis.

**Results:**

We identified compound heterozygous *CDH23* mutations in hearing-loss genes of two families. These include two previously reported pathological mutations, p.Pro240Leu and p.Glu1595Lys, as well as one novel mutation, p.Asn342Ser. The p.Pro240Leu mutation was found in both families. We also identified 26 non-synonymous variants in *CDH23* coding exons from 16 hearing-loss patients and 30 Korean exomes.

**Conclusion:**

The present study is the first to show that *CDH23* mutations cause hearing loss in Koreans. Although the precise contribution made by such mutations needs to be determined using a larger patient cohort, our data indicate that mutations in the *CDH23* gene are one of the most important causes of non-syndromic hearing loss in East Asians. Further exome sequencing will identify common mutations or polymorphisms and contribute to the molecular diagnosis of, and development of new therapies for, hereditary hearing loss.

## Background

Hearing loss is one of the common heterogeneous disorders. Genetic factors account for more than 50% of cases of congenital hearing loss, where the majority of cases exhibit autosomal recessive inheritance [1]. To date, more than 100 mapped loci have been reported, and 55 non-syndromic hearing loss genes have been identified (http://hereditaryhearingloss.org/).

The gene most commonly involved in hearing loss worldwide is *GJB2*[2], while *SLC26A4* is also frequently involved in congenital hearing impairment. The *GJB2* and *SLC26A4* genes make mutation screening relatively easier, and many studies have focused on only these two genes. However, in many ethnic populations, *GJB2* and *SLC26A4* are responsible for only a small percentage of deafness cases [3], and screening of mutations in a large number of genes simultaneously is difficult. Also, it is near-impossible to identify pathogenic mutations by traditional linkage analysis when DNA is available from only small families.

Mutations in the *CDH23* gene are known to be responsible for both Usher syndrome type ID (USH1D) and non-syndromic hearing loss (DFNB12). To date, more than 50 mutations have been reported in patients with Usher syndrome type I (USH1D) who have congenital hearing loss, retinitis pigmentosa (RP), and vestibular dysfunction. A total of 24 mutations have been reported in patients with non-syndromic hearing loss (DFNB12) [4]. A genotype-phenotype correlation study suggested that USH1D was usually associated with nonsense, whereas DFNB12 with missense mutations [5]. Deafness caused by *CDH23* has been found in many populations worldwide, including African–American, Dutch, European, German, Pakistani, Turkish, and Japanese populations [6]. However, clinical application of *CDH23* mutation detection has lagged because of the size of the gene.

Recent advances in DNA enrichment and next-generation sequencing (NGS) technology have allowed rapid and cost-effective analysis of the causative mutations of human disorders, especially those that are heterogeneous in nature [7]. The techniques are particularly applicable to analysis of small families [8]. In the present study, we applied whole-exome sequencing (WES) to study small Korean families negative for mutations in *GJB2* and *SLC26A4*, and we identified *CDH23* mutations in two families with autosomal recessive non-syndromic hearing loss (ARNSHL).

## Methods

### Patients

We performed whole-exome sequencing on 16 affected individuals from 13 families with recessive nonsyndromic hearing loss (Additional file 1: Figure S1 and Additional file 2: Figure S2). All affected individuals had early onset disease and bilateral severe-to-profound hearing loss without additional symptoms (thus, no vestibular dysfunction was evident), and were members of families that were too small to allow performance of linkage analysis. Informed consent was obtained from all participants, and the Institutional Review Board of the Korea National Institutes of Health (NIH) approved this study. Genomic DNA was extracted from peripheral blood samples using a FlexiGene DNA extraction kit (QIAGEN, Hilden, Germany). Probands from each family were found to be negative for *GJB2* and *SLC26A4* mutations based on Sanger sequencing.

### Whole-exome sequencing

Whole exons were captured on the SeqCap EZ Human Exome Library v2.0 (Roche/NimbleGen, Madison, WI, USA) using 5 μg of genomic DNA. Captured libraries were sequenced using the Solexa GAIIx Genome Analyzer with 78-bp paired-end reads (SR-106) and the Illumina HiSeq 2000 system with 101-bp paired-end reads (SR-209) according to the manufacturers’ protocols. Reads were mapped to the reference human genome (GRCh37, UCSC hg19) using the Burrows-Wheeler Aligner (http://bio-bwa.sourceforge.net/). Single-nucleotide variants (SNVs) and insertions-deletions (indels) were called using SAMtools (http://samtools.sourceforge.net/), based on filtered variants with a mapping quality score of ≥20, and were annotated using ANNOVAR (http://www.openbioinformatics.org/annovar/). Mutations identified by exome sequencing were confirmed by Sanger sequencing and additional control individuals were genotyped with the aid of TaqMan SNP Genotyping Assays.

### In silico analysis

Evolutionary conservation of the sequences and structures of the proteins and nucleic acids was assessed using the ConSeq server (http://conseq.tau.ac.il/). The effect of the identified novel missense mutation was assessed using SIFT (http://sift.jcvi.org), PolyPhen-2 (http://genetics.bwh.harvard.edu/pph2/index.shtml) and MUpro (http://mupro.proteomics.ics.uci.edu/), automatic tools for prediction of the possible impact of an amino acid substitution on the structure and function of a human protein. The 3D molecular structure of the extracellular domain of CDH23 was modeled using I-TASSER (http://zhanglab.ccmb.med.umich.edu/I-TASSER/). Predicted models were found to satisfy with quality criteria, such as C-score and TM-score. Structural analysis and visualization were performed using DeepView/Swiss-PdbViewer (http://spdbv.vital-it.ch/).

## Results

### Whole-exome sequencing

We found compound heterozygous mutations in the *CDH23* from two families, SR-106 and SR-209 by whole exome sequencing (Additional file 1: Figure S1). Table 1 shows the clinical characteristics of the three affected members in these families. All of them have early onset and profound hearing loss (see audiogram in Additional file 1: Figure S1). No patient had any other neurological signs (vestibular dysfunction and vertigo). The results of exome sequencing of the two families are shown in Additional file 3: Table S1. The average number of observed variants per sample was 59,589. Approximately 10,284 variants were identified among the coding non-synonymous variants. Further filtering of common variants using Korean exome data, which included exome data for 30 Koreans from another study [9], and from the Korean genome database TIARA [10] and dbSNP131, reduced to about 529 the number of variants in each sample.

**Table 1:** **Clinical characteristics of three patients in two families with compound heterozygous** ***CDH23*** **mutations**

**Sample**	**Relationship**	Threshold* (Rt) (dB)	Threshold* (Lt) (dB)	**Severity**	Residual hearing in the lower frequencies** (dB)	Hearing in the higher frequencies*** (dB)	**Age**	**Age of awareness**	**Hearing aid/cochlear implant**	**Vertigo**
SR-106		NR at 90 dB nHL	NR at 90 dB nHL	Profound	N.D.	N.D.	3	3	None	N.D.
SR-209		NR at 90 dB nHL	103	Profound	90 (L)	110 (L)	1.9	1.9	CI	ㅡ
SR-209B	Sibling of SR-209	NR at 90 dB nHL	115	Profound	107.3 (L)	NR (L)	1.3	1.3	CI	ㅡ

N.D., Not Determined; CI, cochlear implant.

*Average of 500, 1000, 2000, and 4000 Hz.

**Average of 125, 250, and 500 Hz.

***Average of 2000, 4000, and 8000 Hz.

### Identification of causative mutations

**Figure 1:**
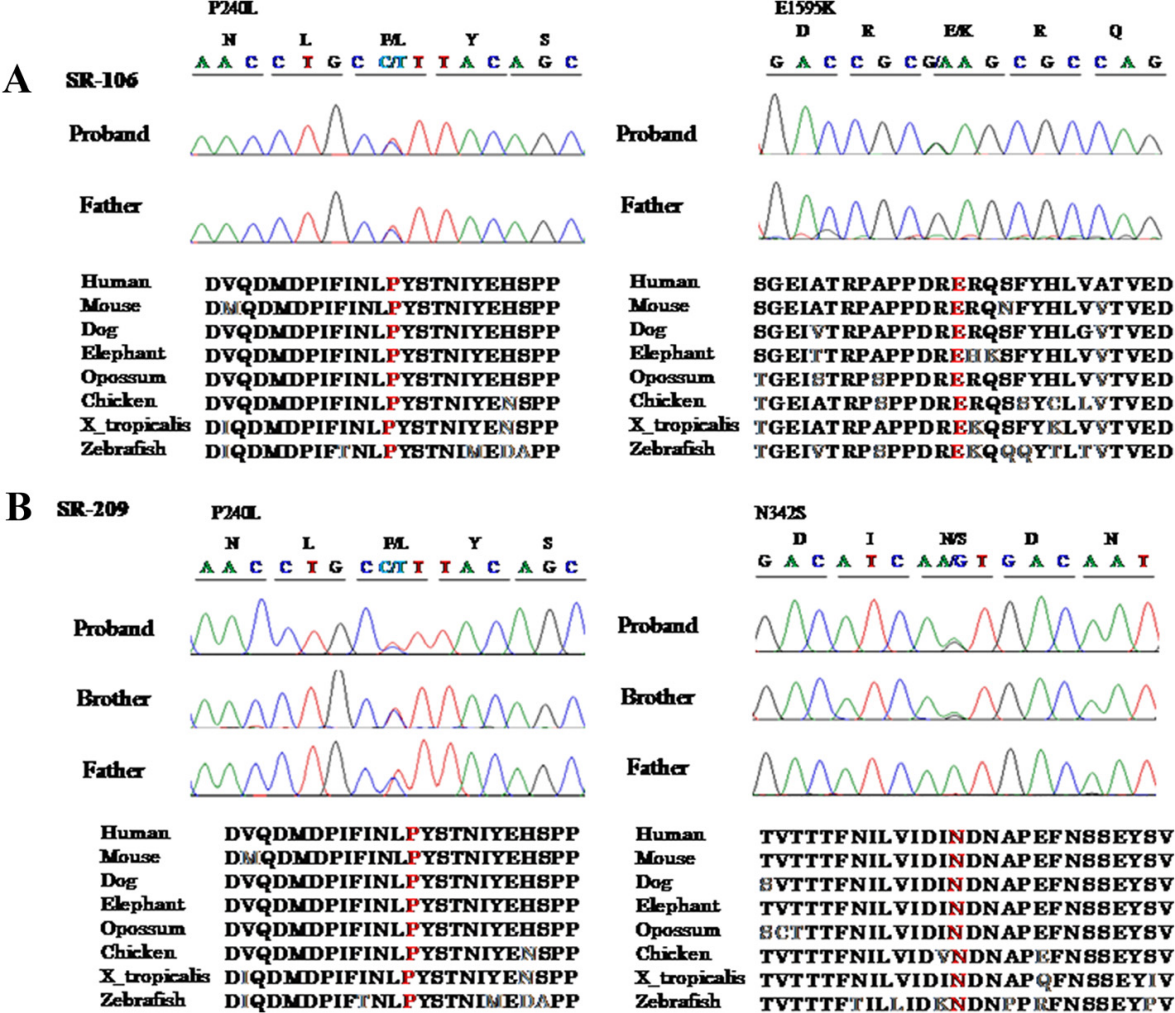
**Confirmation of pathogenic variants in*CDH23*. (A)** Causative mutations were confirmed by capillary sequencing of DNA from the families of affected patients. In family SR-106, compound heterozygous mutations, p.Pro240Leu and p.Glu1595Lys, were identified. One heterozygous p.Pro240Leu mutation was confirmed in the father. **(B)** In family SR-209, compound heterozygous mutations, p.Pro240Leu and p.Asn342Ser, were carried by an affected sibling and a heterozygous p.Pro240Leu mutation by the father. All pathogenic variants occurred at a highly conserved position. The corresponding DNA sequences appear in red.

We focused on 55 known deafness genes (Additional file 3: Table S2), and three to four candidate variants remained in each family. Among them, SR-106 family carried two variants in the *CDH23* gene (Additional file 3: Table S3). Two missense mutations, p.Pro240Leu and p.Glu1595Lys, were identified to be compound heterozygous mutations in the proband of SR-106 family. p.Pro240Leu was confirmed in the patient’s father by Sanger sequencing (Figure 1A). We subsequently sequenced the entire missing region (read depth <5) in all 69 exons of *CDH23*; however, no other mutation was detected. SR-209 family carried two mutations, p.Pro240Leu and p.Asn342Ser in the *CDH23*. Both variants were also found in an affected sibling and p.Pro240Leu was confirmed in the father by Sanger sequencing (Figure 1B). Additionally, we screened for the three mutations mentioned above in 93 Korean patients with hearing loss by Sanger sequencing. Two patients and one patient were heterozygous for p.Pro240Leu (2.15%) and p.Glu1595Lys (1.07%), respectively. Direct sequencing was used to genotype ethnicity-matched negative controls (over 700). The mutations p.Asn342Ser and p.Glu1595Lys were absent in all controls, and p.Pro240Leu was detected in 2 (0.24%) of 818 Korean controls (Table 2).

**Table 2:** ***CDH23*** **mutations detected in this study**

**Nucleotide change**	**Amino acid change**	**Exon**	**Domain**	**Calcium-binding motif**	***In silico*** **analysis**			**Family**	**Controls**	**Reference**
					**SIFT/Score**^**1**^	**PolyPhen-2**^**2**^	**MUpro**^**3**^			
c.C719T	p.Pro240Leu	8	EC3	ㅡ	DAMAGING/0.02	0.999	−1	SR-106, SR-209	2/818	[5]
c.A1025G	p.Asn342Ser	11	EC3	DXNDN	DAMAGING/0	0.905	−1	SR-209	0/707	Novel
c.G4783A	p.Glu1595Lys	36	EC15	DRE	DAMAGING/0	0.987	−1	SR-106	0/707	[6]

^1^Prediction of a change being damaging (<0.05) or tolerated.

^2^Prediction of a change being damaging (>0.85), possibly damaging (0.15-0.85) or benign (<0.15).

^3^Prediction of a change decreasing protein stability (<0) or increasing protein stability (>0) (confidence score between −1 and 1).

### In silico analysis of CDH23 mutations

**Figure 2:**
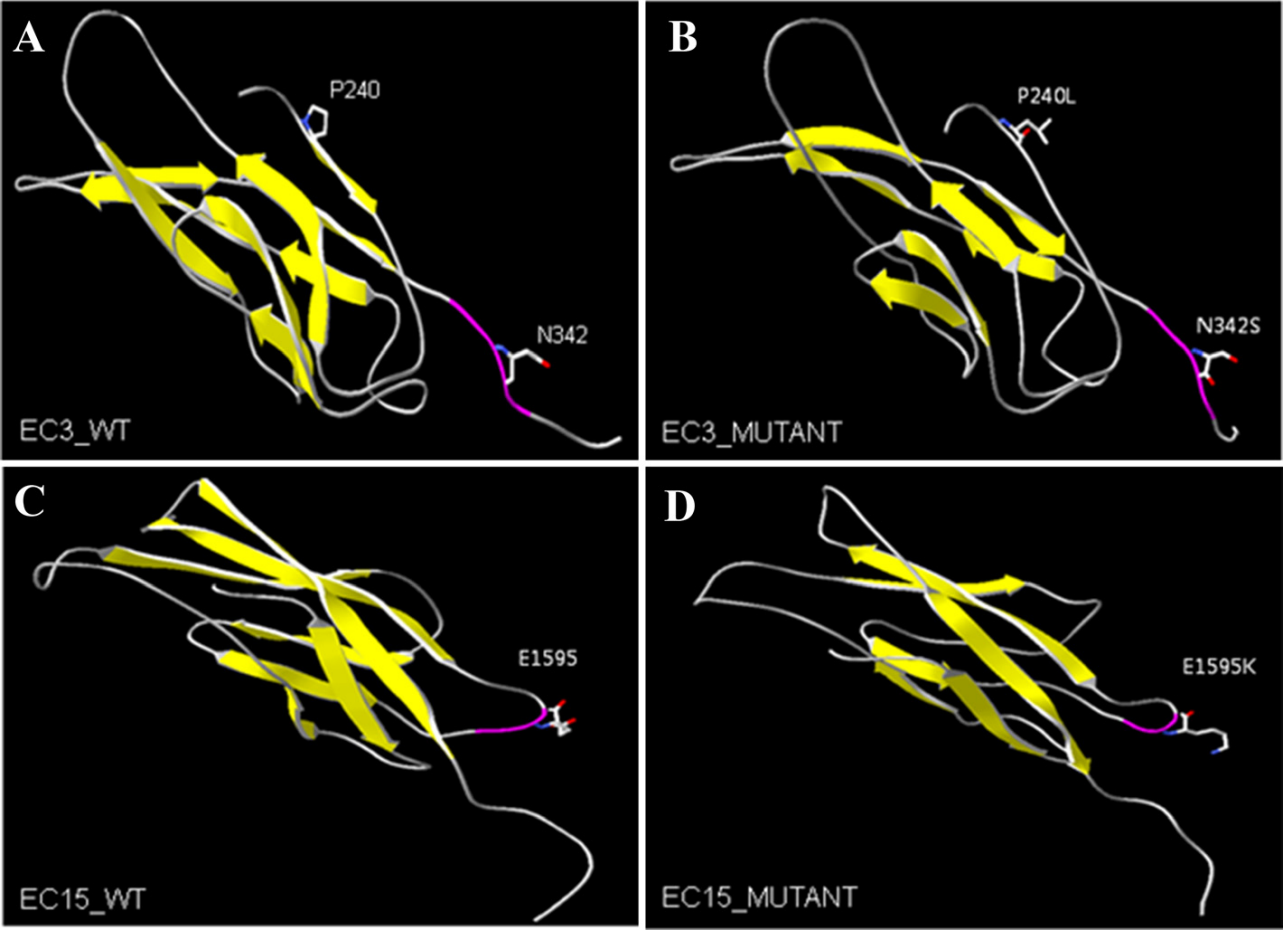
**Molecular modeling of*CDH23*extracellular domains 3 and 15.** Cadherin-23 contains conserved extracellular cadherin (EC) repeat domains. Overall, the structure of all predicted 3D models reveals a typical folding pattern with several beta strands (yellow). **(A, B)** Amino acid residues Pro240 and Asn342 are located in EC3. **(C, D)** Amino acid residue Glu1595 is located in EC15. The structures of A and C are wild-type and those of B and D are mutant. The p.Asn342Ser in EC3 and p.Glu1595Lys in EC15 are associated with highly conserved EC calcium-binding sites (purple) in the linker region between cadherin repeats.

We identified three *CDH23* mutations, comprising two previously reported pathological mutations, p.Pro240Leu and p.Glu1595Lys, and one novel mutation, p.Asn342Ser (Table 2). The ConSeq server revealed that all of the causative mutations were located at a well-conserved site (Figure 1). Figure 2 shows the locations of these mutations in a 3D model of the extracellular cadherin (EC) domains. Mutated EC domains containing residues Asn342 and Glu1595 are part of the highly conserved calcium-binding motifs (LDRE and DXNDN) of EC3 and EC15, located in the region linking cadherin repeats (Figure 2). Calcium ions are usually bound by six or seven oxygen atoms. Loss of one of these oxygens normally reduces calcium binding below the threshold of detection. The p.Glu1595Lys mutation reduces the numbers of oxygen atoms binding calcium. Such mutations are likely to impair the interaction of *CDH23* either with itself or other proteins. Within the polar and uncharged amino acids themselves, the p.Asn342Ser mutation involves the change from a small and hydrophilic asparagine into a very small and neutral serine. The slight difference in length and bulkiness of the side chain may influence the steric hindrance between neighboring residues [11]. Thus it probably decreases calcium affinity and impairs protein function like p.Glu1595Lys. The p.Pro240Leu mutation does not directly interfere with calcium-binding. However, the distinctive cyclic structure of the proline side chain imparts more conformational rigidity than is afforded by other amino acids. The leucine side chain is longer and more flexible (Figure 2). Thus, the p.Pro240Leu substitution may affect protein stability and, consequently, function. All of alterations were predicted by Polyphen-2 and SIFT to pathogenically affect protein structure or function. MUpro predicted that they decrease protein stability (Table 2).

### Identification of CDH23 polymorphism

In addition to these causative mutations, 26 non-synonymous variants were identified in the *CDH23* coding exons from 16 hearing-loss patients and 30 Korean control exomes (Table 3). Among them, 18 variants were reported, of which eight are novel. They also included four variants (Chr10:73337715, Chr10:73405729, Chr10:73558128, and Chr10:73567365) that have potential pathogenicity. They were found in hearing loss families but were not determined to be causative genes in this study.

**Table 3:** **Non-synonymous** ***CDH23*** **mutations considered to be non-causative variants in 16 hearing-loss patients and 30 Korean exomes**

	**Genomic positions (hg19)**	**Exon**	**Nucleotide change**	**Amino acid change**	**dbSNP135_full**	**Deaf samples (16)**	**Korean control exomes**^**1**^	**Reference**
1	Chr10:73337715	9	c.798delA	p.Gly266fs	.	1 (SR-255S)	0/128	Novel
2	Chr10:73377097	11	c.G1081T	p.Ala361Ser	.	.	1/30	Novel
3	Chr10:73405717	12	c.G1270A	p.Val424Met	rs2305207	.	1/30	rs2305207
4	Chr10:73405729	12	c.G1282A	p.Asp428Asn	rs188376296	1 (SR-68)	2/128	rs188376296
5	Chr10:73434888	14	c.G1469C	p.Gly490Ala	rs1227049	8	14/30	[5]
6	Chr10:73434906	14	c.G1487A	p.Ser496Asn	rs10999947	7	54/128	[5]
7	Chr10:73464825	24	c.G2891A	p.Arg964Gln^2^	.	1 (SR-153)	0/128	Novel
8	Chr10:73466729	25	c.G3029A	p.Arg1010His	.	.	1/30	Novel
9	Chr10:73472553	27	c.G3352A	p.Gly1118Ser^2^	.	1 (SR-931)	0/328	Novel
10	Chr10:73492032	31	c.T4004C	p.Val1335Ala	.	.	1/30	Novel
11	Chr10:73492079	31	c.A4051G	p.Asn1351Asp	rs1227065	16	29/30	[5]
12	Chr10:73498355	33	c.G4310A	p.Arg1437Gln	rs56181447	5	8/30	[5]
13	Chr10:73501595	36	c.C4762T	p.Arg1588Trp	.	.	1/30	[5]
14	Chr10:73501556	36	c.G4723A	p.Ala1575Thr	rs1227051	16	28/30	[5]
15	Chr10:73537614	37	c.G5023A	p.Val1675Ile	rs17712523	8	7/30	[5]
16	Chr10:73544086	40	c.G5411A	p.Arg1804Gln	rs3802711	10	16/30	[5]
17	Chr10:73544093	40	c.C5418G	p.Asp1806Glu	rs74145660	3	6/30	[5]
18	Chr10:73550117	44	c.C5996G	p.Thr1999Ser	rs11592462	6	8/30	[5]
19	Chr10:73550969	45	c.G6130A	p.Glu2044Lys	rs10466026	12	26/30	[5]
20	Chr10:73558128	48	c.G6847A	p.Val2283Ile^3^	rs41281334	1 (SR-1016)	3/128	[5]
21	Chr10:73558886	49	c.G7073A	p.Arg2358Gln	rs4747194	12	26/30	[5]
22	Chr10:73558952	49	c.C7139T	p.Pro2380Leu	rs4747195	12	26/30	[5]
23	Chr10:73562763	52	c.A7591G	p.Met2531Val	.	.	1/30	Novel
24	Chr10:73567365	57	c.T8401G	p.Phe2801Val^3^	rs3802707	.	2/30	[5]
25	Chr10:73571307	62	c.G9238A	p.Ala3080Thr	.	1 (SR-1016)	3/128	Novel
26	Chr10:73571765	64	c.T9373C	p.Phe3125Leu	rs45583140	2	15/30	rs45583140

^1^Exome data for 30 or 128 Koreans from another study.

^2^Rule out non-segregation.

^3^Reported as a variant with uncertain pathogenic.

## Discussion

We identified three *CDH23* mutations, p.Pro240Leu, p.Glu1595Lys, and p.Asn342Ser, in 2 (15%) of 13 Korean families with ARNSHL by whole-exome sequencing. The present report is the first to demonstrate that *CDH23* is an important causative gene for ARNSHL in Korean patients. Additionally, we screened for eight mutations, including the three (p.Pro240Leu, p.Asn342Ser and p.Glu1595Lys) detected in the present study, and five others (p.Arg301Gln, p.Glu956Lys, p.Arg1417Trp, p.Gln1716Pro, and p.Arg2029Trp) that occur at relatively high frequencies (patient allele frequency > 0.1) in Japanese patients [4], in 93 unrelated Korean hearing loss patients. Two patients and one patient, respectively, were heterozygous for p.Pro240Leu and p.Glu1595Lys, and no other mutation was detected. p.Pro240Leu was reported in Japanese families with ARNSHL as a compound heterozygous or homozygous mutation [5]. Miyagawa *et al*. reported that mutations of the *CDH23* gene are important causes of non-syndromic hearing loss and that p.Pro240Leu accounted for nearly 43.3% (45/105) of all *CDH23*-mutated families in Japanese [4]. In this study, both families with ARNSHL caused by *CDH23* mutations carried the p.Pro240Leu mutation and, additionally, two of 93 hearing-loss patients were heterozygous for the mutation. Thus, p.Pro240Leu is the most common cause of *CDH23*-associated ARNSHL in Asian populations. p.Glu1595Lys was present in 1 of 93 patients. Astuto *et al.* reported that ~5% of recessive non-syndromic hearing loss might be caused by mutation of *CDH23*[6]. Although *GJB2* and *SLC26A4* mutations were absent in our patients, our results show that *CDH23* mutation caused ARNSHL in 2 of 13 (15%) affected Korean families and that 3 of 93 patients with hearing loss carried a heterozygous mutation. The precise frequency will be determined by future mutation analysis of a large patient cohort, but is estimated to be high. Therefore, *CDH23*, as well as *GJB2* and *SLC26A4,* should be included in screening.

*CDH23* (NM_22124) has 69 exons and encodes cadherin 23, a protein of 3,354 amino acids with 27 EC domains, a single transmembrane domain, and a short cytoplasmic domain [12]. It is a putative calcium-dependent adhesion molecule required for proper morphogenesis of hair bundles of inner ear neurosensory cells. Mutations in *CDH23* cause the stereocilia of hair cells in the inner ear of Waltzer mice (a model of *USH1D*) to become disorganized [13,14]. Many deafness mutations in CDH23 are in the calcium-binding motif of the linker region between EC repeats. All mutations detected in the present study were missense in nature, and in the EC domain. Of the three mutations, two (p.Glu1595Lys and p.Asn342Ser) affected highly conserved EC calcium-binding sites (Figure 2B and D). The sites are LDRE and DXNDN, and are thought to be essential for linearization, rigidification, and dimerization of CDH23 [15,16]. The p.Glu1595Lys mutation may disrupt a conserved LDRE calcium-binding motif in the fifteenth EC domain. These EC domains are involved in cell-to-cell adhesion via hemophilic calcium-dependent interactions [16]. Molecular modeling of the p.Glu1595Lys mutation shows impairment of calcium binding [17]. Since calcium provides rigidity to the elongated structure of cadherin molecules, thereby enabling hemophilic lateral interaction, this mutation is likely to impair interactions of *CDH23* molecules with either *CDH23* or other proteins. Likewise, the p.Asn342Ser mutation is also located in a highly conserved EC calcium-binding site (DXNDN). Thus the mutation probably decreases calcium affinity and impairs protein function. Their pathogenic effects on protein structure or function are supported by *in silico* analysis (Table 2).

Additionally, we identified 26 non-synonymous variants in the *CDH23* coding exons from 16 hearing-loss patients and 30 Korean control exomes (Table 3). Among them, four variants have the potential to be pathogenic. pGly266fs in SR-255S showed co-segregation in the family and was absent in 128 Korean control exomes. However, it was heterozygous, so we could not conclude that it is a causative mutation. p.Asp428Asn in SR-68 was also heterozygous. DNA samples from family members were not available. Thus, we could not confirm its pathogenicity by segregation analysis. p.Val2283Ile and p.Phe2801Val were identified as compound heterozygous mutations in SR-1016. Co-segregation was confirmed by Sanger sequencing of family members, but these two variants were detected in three of the 128 Korean control exomes. Thus, we concluded that these two variants are not causative mutations. However, a heterozygous mutation in a recessive gene may be relevant to the hearing loss phenotype if it coexists with another heterozygous mutation [5], and it is a low frequency in the general Korean population might not rule out the possibility that it is pathogenic. Further exome data accumulation and establishment of a database of common mutations in Korean patients with hearing loss will help us to determine whether they are pathogenic or not.

We performed parallel sequencing of the whole exomes of 13 small families and rapidly and successfully identified the *CDH23* mutation in two families. In two of the other 11 families, hearing loss was caused by the *MYO15A* mutation [18], the first report of this mutation in Eastern Asia. Whole-exome sequencing allowed us to screen mutations in a large number of genes at the same time and to detect pathogenic mutations in affected individuals who were not identified by classical genetic studies. However, the causative mutations in nine families have still not been identified. In these families, whole-exome sequencing may have missed some mutations in exons because it did not completely cover the targeted region, which may have had a high GC content or repetitive sequences, or because hearing loss was caused by mutations an as-yet-unknown gene. Our search for deafness genes in the remaining families is ongoing.

## Conclusions

We identified *CDH23* mutations in two of 13 families with ARNSHL by whole-exome sequencing. The exact frequency will need to be determined in a future larger cohort mutation analysis, but it is estimated to be high. Therefore, as well as *GJB2* or *SLC26A4* screening, *CDH23* should also be considered. Our results show that whole-exome sequencing is effective when used to detect causative mutations in heterogeneous patients with hereditary hearing loss. In the future, we anticipate the rapid discovery of hearing loss-related genes by whole-exome sequencing, which will improve understanding of the condition.

## Competing interests

The authors do not have any conflicts of interest, financial or otherwise, to declare.

## Author’s contributions

HMW, MHP and SKK designed the study and wrote the article. HMW, MHP and WGY analyzed the data. HJP and JWS collected the samples and examined the family information. HMW and BYK performed the sequencing analyses. All authors read and approved the final manuscript.

## Pre-publication history

The pre-publication history for this paper can be accessed here:

http://www.biomedcentral.com/1471-2350/15/46/prepub

## Supplementary Material

Additional file 1: Figure S1 Pedigrees of two families with ARNSHL, and audiogram of patient SR-209. (A) Filled symbols in each pedigree represent affected individuals. The proband is indicated by an arrow. Asterisks indicate available samples. The two individuals whose exomes were sequenced are shown in red. (B) Audiogram of patient SR-209. No audiogram is available for SR-106, only ABR data. Click here for file

Additional file 2: Figure S2 Pedigrees of 11 families with ARNSHL. All families comprised normally hearing parents and two affected siblings. Asterisks indicate sequenced sample. Two (A and B) of 11 families had causative *MYO15A* mutation [18]. In the other families (C-K), the causative mutations in known deafness genes were not identified. Click here for file

Additional file 3: Table S1 Results of exome sequencing in two individuals with ARNSHL. **Table S2.** List of the 55 deafness genes that were used to filter variants. **Table S3.** Candidate variants identified in this study. Click here for file
