# Supplementary figures and images for: Identification of CDH23 mutations in Korean families with hearing loss by whole-exome sequencing
Source: BMC Med Genet. 2014 Apr 28;15:46. doi: 10.1186/1471-2350-15-46 (PMC4036425; doi:10.1186/1471-2350-15-46)

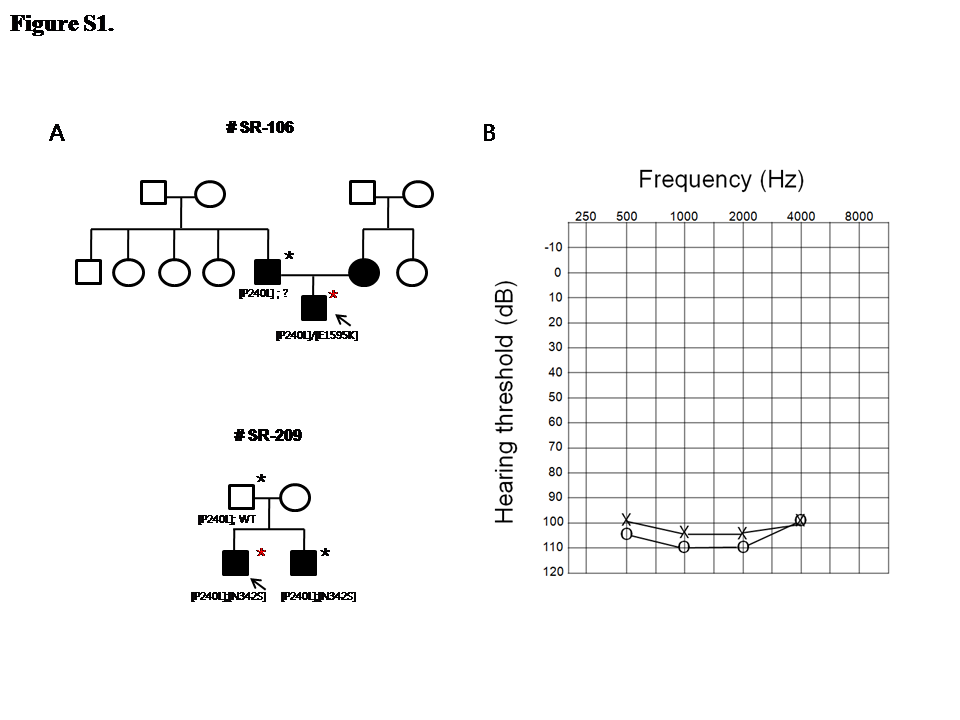

Supplement: Additional file 1: Figure S1 — Pedigrees of two families with ARNSHL, and audiogram of patient SR-209. (A) Filled symbols in each pedigree represent affected individuals. The proband is indicated by an arrow. Asterisks indicate available samples. The two individuals whose exomes were sequenced are shown in red. (B) Audiogram of patient SR-209. No audiogram is available for SR-106, only ABR data. [file 1471-2350-15-46-S1.tiff]

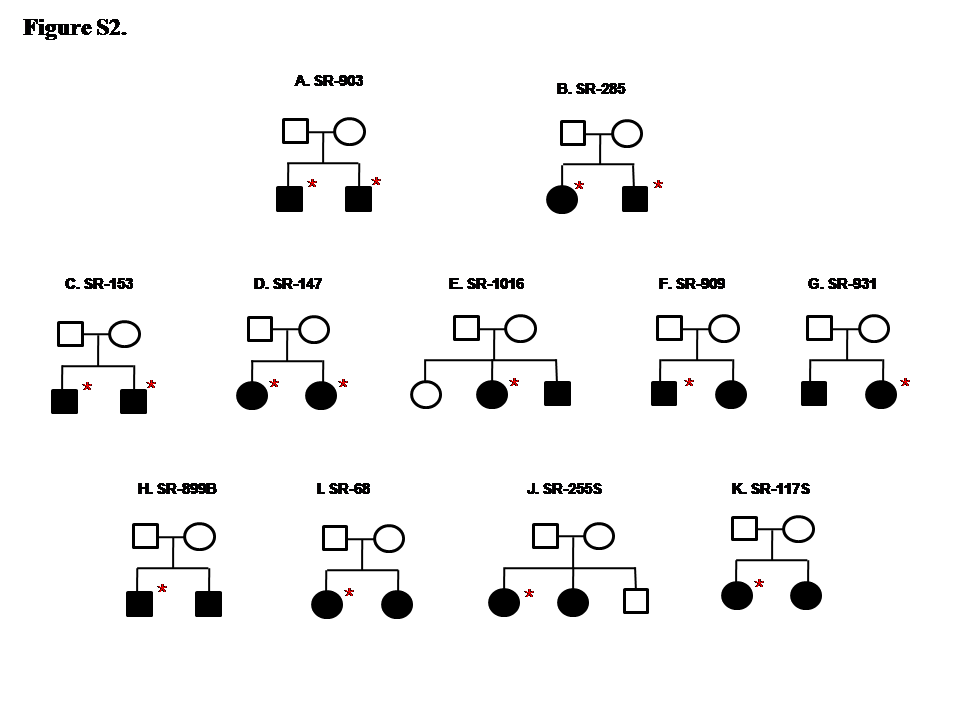

Supplement: Additional file 2: Figure S2 — Pedigrees of 11 families with ARNSHL. All families comprised normally hearing parents and two affected siblings. Asterisks indicate sequenced sample. Two (A and B) of 11 families had causative MYO15A mutation [18]. In the other families (C-K), the causative mutations in known deafness genes were not identified. [file 1471-2350-15-46-S2.tiff]
