# Supplementary material for: Identification of CDH23 mutations in Korean families with hearing loss by whole-exome sequencing
Source: BMC Med Genet. 2014 Apr 28;15:46. doi: 10.1186/1471-2350-15-46 (PMC4036425; doi:10.1186/1471-2350-15-46)
Supplement: Additional file 3: Table S1 — Results of exome sequencing in two individuals with ARNSHL. Table S2. List of the 55 deafness genes that were used to filter variants. Table S3. Candidate variants identified in this study. [file 1471-2350-15-46-S3.pdf]

Table S1. Results of exome sequencing in two individuals with ARNSHL

Parameter	SR-106	SR-209
Total reads	74,947,376	80,339,904
Total yield (bp)	5,845,895,328	8,114,330,304
Mappable reads	69,146,519	68,169,703
Mappable yield (bp)	5,226,122,955	6,673,071,263
On-target reads	39,345,288	43,497,122
On-target yield (bp)	2,343,468,939	3,474,691,342
Coverage of target region (more than 10×)	90%	91%
Mean read depth of targeted region	53.2×	79×
Mean read depth of called variants	47×	52×
Number of total variants	56,508	62,671
Number of coding variants	20,780	20,100
Number of missense, nonsense, splice, and indel variants	10,656	9,912
After Korean control exome* filtering	665	533
After dbSNP131 filtering	579	480(dbSNP135)
Variants in reported deaf genes	3	4
Recessive (at least two variant in a gene)	1	1

* Korean control exome dataset, which includes exome data for 30 Koreans from another study and the Korean genomes database, TIARA.

Table S2. List of the 55 deafness genes that were used to filter variants

	Locus (OMIM)	Location	Gene (OMIM)	Type
1	DFNA20	17q25	*ACTG1*	dominant
2	DFNB73	1p32.3	*BSND*	recessive
3	DFNA44	3q28-29	*CCDC50*	dominant
4	DFNB12	10q21-q22	*CDH23*	recessive
5	DFNB29	21q22	*CLDN14*	recessive
6	DFNA9	14q12-q13	*COCH*	dominant
7	DFNA13,DFNB53	6p21	*COL11A2*	dominant,recessive
8	DFNA5	7p15	*DFNA5*	dominant
9	DFNA1	5q31	*DIAPH1*	dominant
10	DFNA39	4q21.3	*DSPP*	dominant
11	DFNB36	1p36.3	*ESPN*	recessive
12	DFNB35	14q24.1-24.3	*ESRRB*	recessive
13	DFNA10	6q22-q23	*EYA4*	dominant
14	DFNA3A,DFNB1	13q11-q12	*GJB2*	dominant,recessive
15	DFNA2B	1p35.1	*GJB3*	dominant
16	DFNA3B	13q12	*GJB6*	dominant
17	DFNB32	1p13.3-22.1	*GPSM2*	recessive
18	DFNB25	4p13	*GRXCR1*	recessive
19	DFNB39	7q21.1	*HGF*	recessive
20	DFNA2A	1p34	*KCNQ4*	dominant
21	DFNB66/67	6p21.2-22.3	*LHFPL5*	recessive
22	DFNB77	18q12-q21	*LOXHD1*	recessive
23	DFNB63	11q13.2-q13.4	*LRTOMT*	recessive
24	DFNB49	5q12.3-q14.1	*MARVELD2*	recessive
25	DFNA4	19q13	*MYH14*	dominant
26	DFNA17	22q	*MYH9*	dominant
27	DFNB3	17p11.2	*MYO15A*	recessive
28	DFNA48	12q13-q14	*MYO1A*	dominant
29	DFNB30	10p11.1	*MYO3A*	recessive
30	DFNA22,DFNB37	6q13	*MYO6*	dominant,recessive
31	DFNB22	16p12.2	*OTOA*	recessive
32	DFNB9	2p22-p23	*OTOF*	recessive
33	DFNB23	10p11.2-q21	*PCDH15*	recessive
34	DFNB59	2q31.1-q31.3	*DFNB59*	recessive
35	DFNX2 (DFN3)	Xq21.1	*POU3F4*	X-linked
36	DFNA15	5q31	*POU4F3*	dominant
37	DFNX1 (DFN2)	Xq22	*PRPS1*	X-linked
38	DFNB24	11q23	*RDX*	recessive
39	DFNB4	7q31	*SLC26A4*	recessive
40	DFNB61	7q22.1	*SLC26A5*	recessive
41	DFNB16	15q21-q22	*STRC*	recessive
42	DFNA12,DFNB21	11q22-24	*TECTA*	dominant,recessive
43	DFNA28	8q22	*GRHL2*	dominant
44	DFNA51	9q21	*TJP2*	dominant
45	DFNA36,DFNB7/11	9q13-q21	*TMC1*	dominant,recessive
46	DFNB6	3p14-p21	*TMIE*	recessive
47	DFNB8/DFNB10	21q22	*TMPRSS3*	recessive
48	DFNB28	22q13	*TRIOBP*	recessive
49	DFNB79	9q34.3	*TPRN*	recessive

50	DFNB18	11p14-15.1	*USH1C*	recessive
51	DFNA6	4p16.3	*WFS1*	dominant
52	DFNB31	9q32-q34	*DFNB31*	recessive
53	DFNA50	7q32.2	*MIR96*	dominant
54	DFNA11,DFNB2	11q12.3-q21	*MYO7A*	dominant,recessive
55	DFNB84	12q21.2	*PTPRQ*	recessive

Table S3. Candidate variants identified in this study.

	Genomic positions (Hg19)	Gene	Nucleotide change	Protein change	Status
SR-106	Chr2:26707353	*OTOF*	c.T1194A	p.Asp398Glu	
	Chr10:73330641	*CDH23*	c.C719T	p.Pro240Leu	Causative mutation
	Chr10:73501616	*CDH23*	c.G4783A	p.Glu1595Lys	Causative mutation
SR-209	Chr4:88536459	*DSPP*	c.2645_2646insTAGTGACAG	p.Ser882delinsSerSerAspSer	
	Chr10:73330641	*CDH23*	c.C719T	p.Pro240Leu	Causative mutation
	Chr10:73377041	*CDH23*	c.A1025G	p.Asn342Ser	Causative mutation
	Chr12:57431829	*MYO1A*	c.G1785T	p.Gln595His	
